# The impact of digital rural construction on grain production resilience

**DOI:** 10.3389/fnut.2026.1901996

**Published:** 2026-08-05

**Authors:** Xinyu Du

**Affiliations:** School of Economics and Management, Chongqing Normal University, Chongqing, China

**Keywords:** digital rural construction, fiscal support for agriculture, grain production resilience, new quality agricultural productive forces, threshold effect

## Abstract

Ensuring resilient grain production is essential for food availability, price stability, and nutrition security under growing climatic, market, and geopolitical uncertainty. However, limited evidence explains how digital rural construction strengthens resilience specifically at the grain-production stage. Using panel data from 30 Chinese provinces from 2012 to 2023, this study constructs composite indices of digital rural construction and grain production resilience and applies a two-way fixed effects model, a mediation model, and a panel threshold model to examine its direct effect, transmission mechanism, and fiscal boundary condition. The results show that digital rural construction significantly improves grain production resilience, and this conclusion remains robust after sample adjustments, alternative variable measurement, and endogeneity tests. Heterogeneity analysis indicates stronger effects in western China, major grain-producing areas, and production-consumption balanced areas. Mechanism analysis shows that new quality agricultural productive forces partially mediate this relationship by upgrading agricultural labor, production tools, and factor allocation. The threshold results reveal two fiscal-support thresholds, 0.0699 and 0.1236: the effect of digital rural construction is strongest below the first threshold, weakens between the two thresholds, and becomes statistically insignificant above the second threshold. These findings provide policy evidence for strengthening food and nutrition security through digital rural development and more efficient fiscal support.

## 1 Introduction

Global food and nutrition security is increasingly exposed to multiple, overlapping sources of uncertainty. In recent years, climate change, extreme weather events, geopolitical conflicts, fluctuations in international grain prices, and disruptions to global supply chains have shifted the food security agenda from a narrow concern with aggregate output to a broader concern with stable access to sufficient, safe, and nutritious food. The State of Food Security and Nutrition in the World 2024 indicates that global progress toward ending hunger, food insecurity, and all forms of malnutrition remains insufficient and uneven, and that billions of people still lack reliable access to healthy diets (1). For China, a developing country with a population of more than 1.4 billion, resilient grain production is closely related not only to domestic economic stability and basic livelihood protection but also to the affordability and availability of staple foods that underpin dietary energy supply and nutrition security. Unlike ordinary industrial goods, grain production is jointly shaped by natural conditions, resource endowments, factor inputs, technological capacity, and institutional arrangements. It is therefore cyclical, vulnerable, and partly public-good oriented.

Against this background, food security can no longer be understood solely as a static question of output sufficiency. It increasingly depends on whether the production system can maintain stable supply, recover rapidly from shocks, adapt continuously, and support long-term access to nutritionally essential foods. Recent evidence from China’s forest carbon sink research further shows that climate-response capacity is shaped not only by ecological endowments but also by research and development intensity, industrial structure upgrading, governance intervention, and regional spillover effects (2). This reinforces the need to examine grain production resilience within a broader framework that links climate pressure, ecological governance, technological innovation, and food and nutrition security capacity.

At the same time, China has continued to promote the application of digital technologies in agricultural and rural modernization. The Action Plan for Digital Rural Development (2022–2025) emphasizes digital infrastructure upgrading, smart agriculture innovation, and digital governance capacity, with the aim of accelerating the digital transformation of agricultural production and management and achieving initial progress in smart agriculture by 2025 (3). The 2024 Central No. 1 Document further calls for continued implementation of the digital rural development strategy, the development of smart agriculture, the narrowing of the urban–rural digital divide, the construction of regional big data platforms, and stronger coordination and sharing of agricultural information. The National Smart Agriculture Action Plan (2024–2028) also proposes that, by the end of 2028, information technology should play a visible role in reducing costs, increasing yields, and improving efficiency in grain, oil, and other important agricultural products (4). These policy developments suggest that digital rural construction is becoming an important support for risk warning, resource allocation, and system recovery in grain production. The concept of resilience was first used to explain the capacity of ecological systems to maintain structural and functional stability under external disturbance (5). It was later introduced into food systems and agricultural economics to analyze the ability of food systems to sustain supply, nutrition security, and social welfare under shocks and stresses (6, 7). From this perspective, grain production resilience is not a simple extension of output level. Rather, it captures the integrated capacity of a grain production system to ensure stable supply, respond to risks, stabilize food prices, and maintain the production-side foundation for adequate dietary intake in complex and uncertain environments.

Existing studies on grain production resilience have mainly addressed three issues: measurement, spatiotemporal evolution, and determinants. One strand of research focuses on evaluation systems and regional differences, arguing that grain production resilience in China displays clear spatial disparities and is influenced by agricultural resource endowments, technological conditions, stages of economic development, and infrastructure (8). A second strand extends the object of analysis from production alone to the broader food system, emphasizing that food security depends not only on production capacity but also on circulation, consumption, governance, and risk regulation (9). A third strand examines the formation of food resilience from the perspectives of digital inclusive finance, labor migration, and farmland operation, suggesting that financial access, factor mobility, and scale operation affect the stability of food systems and grain production by improving resource allocation (10, 11). These studies provide an important foundation for understanding grain production resilience. However, relatively little attention has been paid to how digital rural construction, as a comprehensive digital and institutional arrangement, may strengthen the production-side basis of food availability, price stability, and nutrition security.

Digital rural construction is a concrete extension of the Digital China strategy to agriculture and rural areas and an important pathway for agricultural and rural modernization. Unlike a narrow focus on information infrastructure, digital rural construction encompasses digital infrastructure, rural industrial digitalization, digital finance, rural e-commerce, digital governance, and the development of agricultural data resources. Its essence lies in embedding digital technologies, data elements, and platform-based organizations into the economic and social operation of rural areas (12). The integration of the digital economy and agriculture can promote agricultural modernization by improving infrastructure, optimizing the behavior of agricultural operators, and reshaping industrial processes (13). Related evidence from Chinese listed firms shows that digital transformation can reduce supply chain concentration by improving information transparency and information acquisition capability, thereby supporting supply-chain diversification and robustness (14). Although this evidence is drawn from the corporate sector, it is consistent with the argument that digital technologies reshape inter-organizational relationships and resource coordination, which is relevant to the resilience of agricultural production and circulation systems. The rural digital economy can also increase agricultural total factor productivity through multiple pathways, including production upgrading, management transformation, and industrial improvement, thereby enhancing the quality of agricultural development (15). International research similarly shows that smart farming and digital agriculture can improve agricultural efficiency and sustainability through precision management, resource conservation, risk identification, and knowledge sharing (16, 17). Taken together, these findings indicate that digital technology is moving from an external auxiliary tool to a key input in the agricultural production function, with deeper implications for production, management, governance, and food-system resilience.

From the perspective of grain production resilience, digital rural construction may operate through at least three channels that are relevant to nutrition-oriented food security. First, digital infrastructure improves rural information accessibility and alleviates information asymmetry in traditional grain production. This enables agricultural operators to obtain meteorological information, input prices, market demand, and policy support in a more timely manner, thereby improving risk anticipation and production decision-making. More stable grain production can reduce the likelihood of supply disruption and sharp price fluctuations in staple foods, which are important determinants of household food access. Second, industrial digitalization introduces intelligent agricultural machinery, remote sensing, the agricultural Internet of Things, agricultural big data, and digital extension services into grain production. These technologies improve precision in sowing, irrigation, plant protection, harvesting, and post-disaster recovery. Smart agriculture can strengthen the shock resistance and recovery capacity of grain production systems through intelligent forecasting, disaster identification, and factor allocation (18). Third, digital finance, rural e-commerce, and digital governance tools can improve farmers’ financing capacity, market connectivity, and access to public services, thereby enhancing the adaptability of grain production systems under market fluctuations and disaster shocks. Previous research has shown that digital rural construction strengthens the resilience of China’s food system, with rural digital finance and digital service platforms playing relatively stable enabling roles (19). Incorporating digital rural construction into the analytical framework of grain production resilience therefore helps explain how the upstream production capacity required for food and nutrition security is formed in the era of digital transformation.

It is also important to recognize that the effect of digital rural construction on grain production resilience may not be purely linear. On the one hand, the extent to which digital technologies empower agriculture depends on whether productive forces can be upgraded. As the concept of new quality productive forces is extended to agriculture, increasing emphasis has been placed on the coordinated advancement of scientific and technological innovation, data elements, intelligent equipment, and human capabilities. New quality agricultural productive forces are dynamic and regionally differentiated, and their formation is closely associated with agricultural technological progress, the upgrading of production tools, and the reorganization of production factors (20). The digital economy can enhance grain production resilience through technological innovation capacity and agricultural product marketing capacity, while digital inclusive finance can strengthen grain production resilience through agricultural technological innovation (21, 22). This suggests that digital rural construction does not merely improve grain production conditions directly. It may also enhance the stable supply capacity and risk response capacity of grain production systems by upgrading agricultural labor, means of production, and objects of labor. At the same time, the role of digital rural construction is constrained by public resource allocation. Fiscal support for agriculture can provide public investment for agricultural infrastructure, extension services, and risk-prevention systems, all of which are closely related to the capacity of local food systems to maintain stable supply under shocks. Yet if fiscal resources are inefficiently allocated or poorly structured, the enabling effect of digitalization may weaken. Local fiscal expenditure on agriculture, forestry, and water affairs is therefore an important condition for digitalization to strengthen agricultural resilience, and the effect of agricultural informatization on agricultural total factor productivity may also exhibit threshold characteristics (23, 24).

Overall, the existing literature provides a useful foundation for studying digital rural construction and grain production resilience, but three gaps remain. First, studies of digital rural construction have largely focused on high-quality agricultural development, agricultural total factor productivity, farmers’ income, and rural governance, while systematic evidence on its impact on grain production resilience remains limited. Second, research on food resilience often adopts a food-system or supply-chain perspective, leaving the stability, adaptability, and transformative capacity of the production stage underexplored, even though production shocks are transmitted downstream to food availability, affordability, and diet quality. Third, the mechanisms and boundary conditions through which digital rural construction affects grain production resilience have not been sufficiently clarified, particularly the roles of agricultural productivity upgrading and fiscal support for agriculture.

To address these gaps, this study uses panel data from 30 Chinese provinces from 2012 to 2023 to examine how digital rural construction affects grain production resilience. After constructing composite indices of digital rural construction and grain production resilience, this study applies a two-way fixed effects model, a mediation model, and a panel threshold model to identify the direct effect, transmission mechanism, and fiscal boundary condition of digital rural construction. The study contributes to the literature in three respects. First, while existing research has mainly examined food-system resilience or the broader impact of the digital economy on agriculture, this study focuses specifically on the grain-production stage, thereby clarifying how digital rural construction contributes to the stability, recovery, and adaptive capacity of staple-food production. This provides production-side evidence for understanding nutrition security, especially the availability and affordability of staple foods in developing-country contexts. Second, beyond estimating the average effect of digital rural construction, this study introduces new quality agricultural productive forces as a mechanism variable, providing evidence that rural digital transformation enhances grain production resilience by upgrading agricultural laborers, production tools, and factor allocation. Third, by incorporating fiscal support for agriculture as a threshold variable, this study identifies the public-finance conditions under which digital rural construction is more or less effective, offering policy insights into how fiscal support can be better aligned with digital rural development to strengthen grain production resilience and, ultimately, the upstream foundation of food and nutrition security.

## 2 Theoretical analysis and research hypotheses

### 2.1 Direct effect of digital rural construction on grain production resilience

Grain production resilience refers to the comprehensive capacity of a grain production system to maintain stable supply, recover rapidly, and continue adapting and upgrading under multiple shocks, including natural disasters, market fluctuations, resource constraints, and changes in the institutional environment. Drawing on digital technologies, digital platforms, and data elements, digital rural construction systematically reshapes agricultural production, operation, service provision, and governance. It thus provides new technological and organizational foundations for improving grain production resilience. The key to digitally enabled high-quality agricultural development is to overcome the constraints of inadequate information, inefficient resource allocation, and weak risk response in traditional agriculture through information sharing, technology diffusion, and factor reconfiguration (25, 26).

First, digital rural construction can strengthen the risk-resistance capacity of grain production systems. Grain production is highly dependent on natural conditions and is characterized by strong seasonality. Weather changes, the spread of pests and diseases, fluctuations in input prices, and shifts in market demand can all affect production stability. Traditional agricultural operators often rely on experience-based judgment and have limited access to information, making it difficult to identify external shocks in a timely manner. By expanding rural broadband networks, mobile communication, agricultural information platforms, digital financial services, and digital extension services, digital rural construction improves agricultural operators’ access to meteorological, market, input, policy, and technical information. More efficient information transmission enables grain producers to adjust planting schedules, input use, and risk-prevention strategies more quickly, thereby enhancing system stability under external disturbance.

Second, digital rural construction can improve the recovery and adjustment capacity of grain production systems. Resilience is reflected not only in pre-shock protection but also in post-shock recovery. The introduction of remote sensing, the agricultural Internet of Things, intelligent machinery, drone-based field inspection, and digital extension services can improve the speed of disaster identification, machinery dispatch, input delivery, and technical support. The development of digital finance, rural e-commerce, and agricultural socialized service platforms can also ease the financial, market, and service constraints faced by agricultural operators during post-disaster recovery. Digital technology-driven high-quality agricultural and rural development is not simply the replacement of one technology by another; rather, it involves the overall upgrading of production organization, public services, and governance capacity (27). In this sense, digital rural construction can shorten the time required for a grain production system to move from a shocked state back to a stable state.

Third, digital rural construction can promote structural optimization in grain production systems. A higher level of resilience does not simply mean returning to the original state after a shock; it also implies upgrading production modes under pressure. By incorporating data elements into agricultural production, digital rural construction shifts grain production from experience-based judgment to data-driven decision-making, from extensive input use to precision management, and from single-link optimization to coordinated optimization across production, circulation, finance, and governance. Digital technologies empower agricultural development not only by improving production efficiency but also by strengthening agricultural value-chain coordination and reorganizing business models (28). In the grain sector, this transformation helps improve mechanization, intelligence, and greening, enabling the production system to move from traditional factor-input dependence toward a model driven by technology, data, and organizational coordination. Therefore, this study proposes the following hypothesis:

*H1*: Digital rural construction significantly enhances grain production resilience.

### 2.2 Mediating role of new quality agricultural productive forces

The impact of digital rural construction on grain production resilience is not limited to the direct embedding of digital technologies into agricultural production. More fundamentally, it reflects a transformation in agricultural productive forces. New quality agricultural productive forces emphasize scientific and technological innovation as the leading driver and the optimized combination of laborers, means of production, and objects of labor as the foundation. They promote a shift in agricultural production from traditional factor expansion toward efficiency improvement, quality enhancement, and green transformation. Through technology diffusion, platform connectivity, and the embedding of data elements, digital rural construction provides important conditions for this transformation. The process through which the digital economy drives agricultural modernization is, in essence, a process of continuous integration between digital technologies and agricultural production factors, business organizations, and industrial systems (29).

On the one hand, digital rural construction can improve the capabilities of agricultural laborers. Once digital technologies enter the production process, agricultural operators need stronger abilities in information recognition, technology application, and business decision-making. Online agricultural training, agricultural information platforms, rural e-commerce systems, and digital financial tools help farmers, family farms, cooperatives, and agribusinesses obtain production technologies, market information, and policy resources more conveniently. This process supports the transformation of traditional agricultural laborers into new agricultural actors with digital literacy, management capacity, and innovation awareness. As the capabilities of agricultural operators improve, they can respond more actively to disaster shocks, price fluctuations, and resource constraints through production adjustment, technology adoption, and management decisions, thereby enhancing the proactive adaptability of grain production systems.

Moreover, digital rural construction can upgrade both the means and the objects of agricultural production. New production tools, including intelligent agricultural machinery, agricultural sensors, drones, BeiDou navigation, smart irrigation, and agricultural big data platforms, improve precision in sowing, fertilization, irrigation, plant protection, and harvesting. At the same time, data resources, digital credit, agricultural information, and ecological and environmental data are becoming important inputs in agricultural production and management. Digital rural construction can improve agricultural green total factor productivity through technological progress and more efficient resource allocation, thereby shifting agricultural production from traditional input expansion to efficiency improvement and green transformation (30). This process of upgrading intelligent production tools and expanding production objects essentially transforms agricultural productive forces from a traditional form to a more advanced form.

Once new quality agricultural productive forces are formed, they can further strengthen grain production resilience. Intelligent production tools and digital management methods can improve grain production efficiency and reinforce stable supply capacity. Agricultural technological innovation and data-driven decision-making can improve pest and disease monitoring, disaster warning, and post-disaster recovery. Green production technologies and resource-saving management practices can reduce environmental pressure and improve system sustainability. The digital economy promotes urban–rural integration by improving factor allocation efficiency, and such improvement is an important manifestation of the upgrading of agricultural productive forces (31). The county-level digital economy can also promote the development of agricultural socialized services, improve production and management conditions for smallholders through organized service provision, and enhance the overall coordination capacity of agricultural production systems (32). Accordingly, digital rural construction can strengthen the stability, recoverability, and sustainability of grain production systems by transforming agricultural productive forces. This reasoning leads to the second hypothesis:

*H2*: Digital rural construction indirectly enhances grain production resilience by improving new quality agricultural productive forces.

### 2.3 Threshold role of fiscal support for agriculture

Whether digital rural construction can be translated into grain production resilience also depends on public resource allocation and the institutional support environment. Fiscal support for agriculture reflects the extent to which local governments support agricultural and rural development. It is directly related to agricultural infrastructure, extension services, public service provision, and risk-prevention systems. For digital rural construction, fiscal resources can provide funding for rural digital infrastructure, smart agricultural equipment, and agricultural information service platforms. They can also improve the application efficiency of digital technologies in grain production through extension services, subsidies for agricultural socialized services, and disaster relief systems.

When fiscal support for agriculture is relatively low, agricultural infrastructure and public service systems are often weak, and grain producers face high costs in information search, technology adoption, and risk management. In this situation, digital rural construction can compensate for information, technology, and service shortcomings to a considerable extent, leaving substantial room for marginal improvement in grain production resilience. As fiscal support increases, agricultural infrastructure, extension systems, and risk-protection mechanisms gradually improve. Digital rural construction then receives stronger resource and institutional support, making its positive effect on grain production resilience easier to realize.

However, more fiscal support does not automatically produce better outcomes. If fiscal funds are excessively concentrated in general subsidies or traditional projects rather than being effectively directed toward digital infrastructure, agricultural technological innovation, and grain risk-prevention systems, resource misallocation and input redundancy may occur. The key issue is therefore not only the scale of fiscal expenditure but also its structure and allocation efficiency. Both the size and the share of fiscal support for agriculture can affect agricultural efficiency, but their effects depend on whether fiscal resources are effectively transformed into productivity gains. Agricultural subsidies may weaken policy performance if they fail to promote scale operation and efficiency improvement (33). Thus, once fiscal support reaches a certain level, insufficient efficiency in the use of funds may weaken the marginal effect of digital rural construction on grain production resilience. Based on this reasoning, this study proposes the third hypothesis:

*H3*: Fiscal support for agriculture has a threshold effect in the relationship between digital rural construction and grain production resilience.

**Figure 1:**
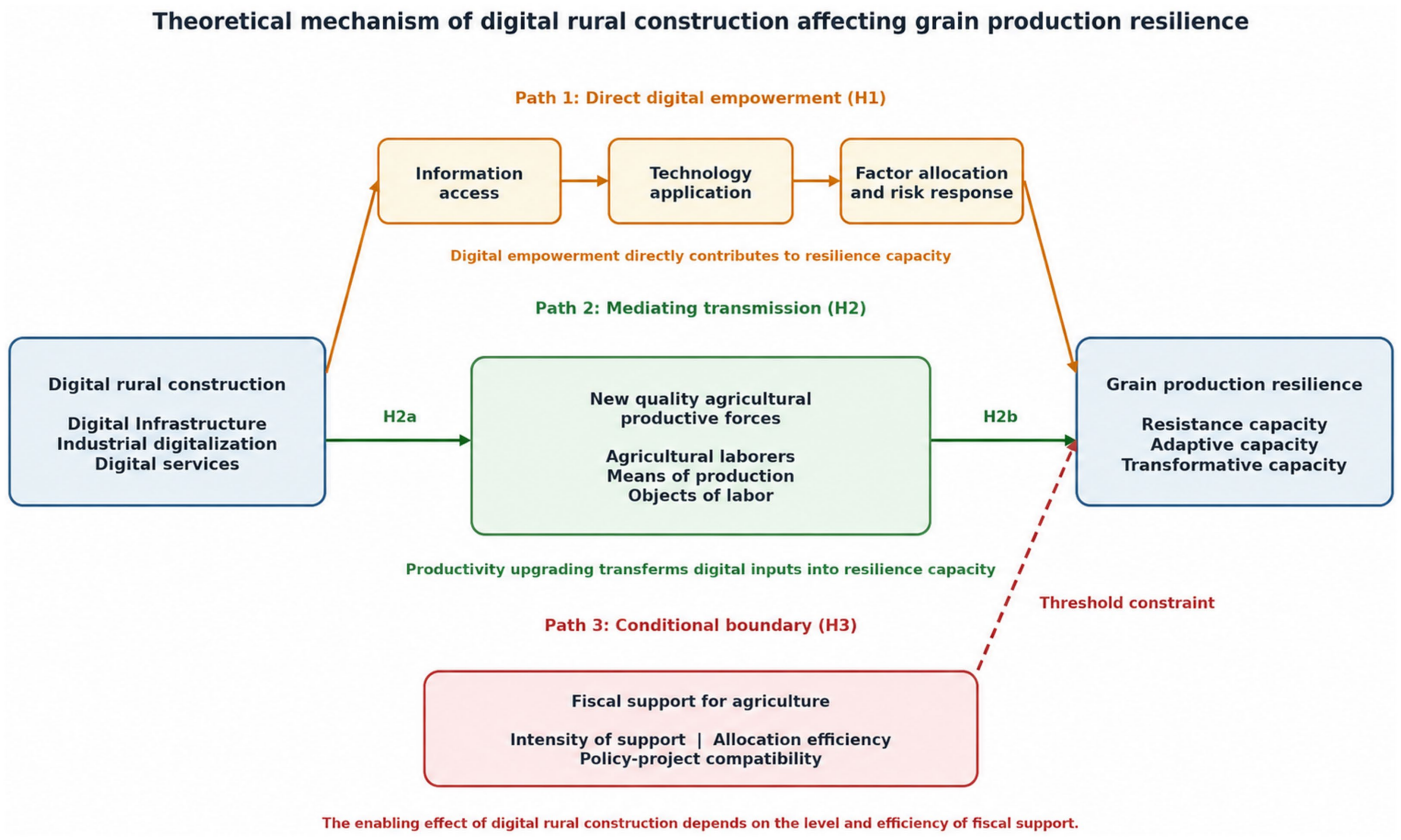
Theoretical mechanism of digital rural construction affecting grain production resilience.

The theoretical mechanism described above is summarized in Figure 1, which illustrates the direct effect, the mediating role of new quality agricultural productive forces, and the threshold constraint of fiscal support for agriculture.

## 3 Research design

### 3.1 Data sources

This study uses panel data for 30 Chinese provinces, municipalities, and autonomous regions from 2012 to 2023, excluding Hong Kong, Macao, Taiwan, and Tibet. The data used to construct the digital rural construction index are obtained from the National Bureau of Statistics of China, the 2023 China Taobao Village Research Report, the Ministry of Agriculture and Rural Affairs, the China Tertiary Industry Statistical Yearbook, and the Peking University Digital Financial Inclusion Index (2012–2023). Data for other variables are mainly drawn from the China Statistical Yearbook, China Agricultural Yearbook, China Rural Statistical Yearbook, China Energy Statistical Yearbook, the National Bureau of Statistics, and provincial statistical yearbooks. To ensure data completeness and usability, missing values for individual years are filled using linear interpolation.

### 3.2 Variable measurement

Dependent variable: grain production resilience (GPR). Compared with resilience at the level of the food system as a whole, grain production resilience has a more specific focus. It refers to the capacity of the grain production stage to continue operating under external shocks, such as natural disasters, market volatility, and policy intervention; restore stability rapidly; and move toward a higher level of development through adaptation and transformation. Given its multidimensional nature, this study constructs an evaluation system based on previous research (10, 19). The index is developed from three dimensions: resistance capacity, adaptive capacity, and transformative capacity. The entropy method is then used to measure grain production resilience in order to improve the objectivity of the results. The evaluation system is shown in Table 1.

**Table 1:** Evaluation system for grain production resilience.

First-level indicator	Second-level indicator	Third-level indicator	Definition	Direction
Resistance capacity	Internal stability	Per capita cultivated land area	Total cultivated land area/agricultural employment (hm2/person)	+
		Share of effectively irrigated area	Effectively irrigated area/crop sown area (%)	+
		Grain structure	Grain structure (%)	+
		Urban employees in agriculture, forestry, animal husbandry, and fishery	Number of urban employees in agriculture, forestry, animal husbandry, and fishery	+
	Production-supply robustness	Per capita grain output	Per capita grain output (kg/person)	+
		Grain consumer price index	Current grain price/base-period grain price (%)	−
		Grain yield per unit area	Grain output/grain sown area (kg/hm2)	+
Adaptive capacity	Sustainability	Fertilizer use per unit area	Fertilizer use/crop sown area (kg/hm2)	−
		Pesticide use per unit area	Pesticide use/crop sown area (kg/hm2)	−
		Agricultural film use per unit area	Agricultural film use/crop sown area (kg/hm2)	−
	Recoverability	Multiple cropping index	Total crop sown area/cultivated land area (%)	+
		Crop disaster rate	Disaster-affected area/sown area (%)	−
		Import and export dependence	Import and export dependence (%)	+
		Per capita electricity consumption	Rural electricity consumption/rural population (kWh/person)	+
Transformative capacity	Diversified coordination	Total machinery power per unit area	Total machinery power per unit area (kW/hm2)	+
		Fixed-asset investment in agriculture, forestry, animal husbandry, and fishery	Fixed-asset investment in agriculture, forestry, animal husbandry, and fishery (100 million yuan)	+
		Value added of agriculture, forestry, animal husbandry, and fishery	Value added of agriculture, forestry, animal husbandry, and fishery (100 million yuan)	+
	Technological progress	Agricultural R&D expenditure	Agricultural R&D expenditure (100 million yuan)	+
		Number of technical personnel	Agricultural technical personnel/rural population (persons/10,000 persons)	+
		Local fiscal expenditure on science and technology	Local fiscal expenditure on science and technology (100 million yuan)	+

Core explanatory variable: digital rural construction (DRC). Digital rural construction refers to the systematic integration of digital technologies, such as the Internet, big data, and artificial intelligence, into all areas of rural economic and social development. It promotes the comprehensive digital transformation of rural production, daily life, and governance. Following Zhang et al. (34), this study constructs a composite evaluation system for digital rural construction using nine indicators from three dimensions: digital infrastructure, industrial digitalization, and digital services. These indicators are intended to capture the overall development level of digital rural construction, as shown in Table 2.

**Table 2:** Evaluation system for digital rural construction.

First-level indicator	Indicator	Definition and measurement	Unit	Direction	Source
Digital infrastructure	Internet accessibility	Number of rural broadband access households	10,000 households	+	National Bureau of Statistics
	Fixed digital-device accessibility	Number of computers per 100 rural households	Units	+	National Bureau of Statistics
	Mobile digital-device accessibility	Number of mobile phones per 100 rural households	Units	+	National Bureau of Statistics
Industrial digitalization	Sales digitalization	Share of Taobao villages in administrative villages	%	+	China Taobao Village Research Report
	Digitalization of agricultural production	Ratio of national modern agricultural demonstration zones and industrial parks to county-level administrative units	−	+	Ministry of Agriculture and Rural Affairs; National Bureau of Statistics
	Digitalization of the financial sector	Digitalization degree index of digital finance	−	+	Peking University Digital Financial Inclusion Index
Digital services	Digital financial services	Coverage breadth index of digital finance	−	+	Peking University Digital Financial Inclusion Index
	E-commerce services	Weekly delivery frequency in rural areas	Times	+	China Tertiary Industry Statistical Yearbook
	Mobile payment level	Mobile payment index	−	+	Peking University Digital Financial Inclusion Index

Mechanism variable: new quality agricultural productive forces (NQAFP). Drawing on studies of new quality agricultural productive forces (20, 35, 36), this study follows the logic of the three core elements of agricultural production and constructs a composite evaluation system from the dimensions of agricultural laborers, objects of labor, and means of production. The resulting composite index is used as the mechanism variable, as shown in Table 3. Digital rural construction can facilitate the optimized allocation of rural production factors and drive the transformation and upgrading of agricultural production modes, thereby expanding new quality agricultural productive forces. In turn, new quality agricultural productive forces can improve grain production resilience by increasing agricultural total factor productivity and strengthening risk resistance and stable production capacity.

**Table 3:** Evaluation system for new quality agricultural productive forces.

Target layer	Criterion layer	Primary indicator	Secondary indicator	Measurement	Direction
New quality agricultural productive forces	Agricultural laborers	Laborer skills	Education level	Average years of education of rural laborers	+
			Share of rural adult technical training	Graduates from rural adult cultural and technical training schools/rural population	+
		Labor productivity	Per capita output value of the primary industry	Output value of the primary industry/employment in the primary industry	+
			Per capita income of rural residents	Per capita disposable income of rural residents	+
		Employment orientation	Rural labor mobility	Migrant labor/rural employment	−
	Agricultural objects of labor	Ecological environment	Green environmental protection	Forest coverage rate	+
				Fiscal expenditure on environmental protection/government public fiscal expenditure	+
			Pollution control	Share of agricultural COD emissions/output value of the primary industry	−
				Share of agricultural ammonia nitrogen emissions/output value of the primary industry	−
		New-quality industries	Agriculture-related services	Value added of agriculture, forestry, animal husbandry, and fishery services	+
	Agricultural means of production	Material means of production	Traditional infrastructure	Rural road mileage/rural population	+
			Digital infrastructure	Rural broadband access users/rural households	+
				Length of optical cable per square meter	+
			Energy consumption	Per capita rural electricity consumption	+
		Intangible means of production	Scientific and technological innovation	Number of agricultural science and technology workers	+
				Stock of agricultural R&D investment	+
			Digitalization level	Rural digital financial inclusion investment index	+
				Rural digital financial inclusion mobile payment index	+

Threshold variable: fiscal support for agriculture (FSA). This variable is measured by the ratio of local fiscal expenditure on agriculture, forestry, and water affairs to local general public budget expenditure. It reflects the intensity of local government fiscal support for agricultural and rural development.

Control variables. Following the research design and variable selection in related studies (19, 37–39), this study includes the following controls. Urbanization level (URB) is measured by the ratio of the urban population to the resident population. Agricultural mechanization intensity (AMI) is measured by the ratio of total agricultural machinery power to total crop sown area. Human capital level (HCL) is measured by the average years of education of rural residents. Economic development level (EDL) is measured by the natural logarithm of per capita GDP. Traditional infrastructure construction (TIC) is measured by the ratio of rural road mileage to the rural population. Descriptive statistics are reported in Table 4.

**Table 4:** Descriptive statistics.

Variable	Observations	Mean	Std. dev.	Min.	Max.
Digital rural construction	360	0.440	0.128	0.156	0.712
Grain production resilience	360	0.142	0.072	0.058	0.453
Economic development level	360	10.963	0.455	10.086	12.219
Urbanization level	360	0.607	0.117	0.392	0.898
Agricultural mechanization intensity	360	0.655	0.226	0.327	1.279
Human capital level	360	7.733	0.638	6.202	9.490
Traditional infrastructure construction	360	5.461	0.577	3.990	6.447
New quality agricultural productive forces	360	0.169	0.073	0.062	0.534
Fiscal support for agriculture	360	0.113	0.034	0.040	0.204

### 3.3 Model specification

To test whether digital rural construction promotes grain production resilience, this study estimates the following two-way fixed effects model.

$$\mathrm{GP}R_{i,t}=\alpha +\mathrm{\theta DR}C_{i,t}+\beta X_{i,t}+\mu_{i}+\lambda_{t}+\varepsilon_{i,t}$$ (1)

In Equation 1, $\mathrm{GP}R_{i,t}$ denotes grain production resilience in province i in year t, $\mathrm{\theta DR}C_{i,t}$ denotes the level of digital rural construction, and $X_{i,t}$ represents the vector of control variables. Province fixed effects and year fixed effects are denoted by $\mu_{i}$ and $\lambda_{t}$ , respectively, and $\varepsilon_{i,t}$ is the random error term.

To examine the mediating role of new quality agricultural productive forces in the relationship between digital rural construction and grain production resilience, this study follows the two-step mediation approach proposed by Jiang (40) and estimates the following models.

$$\text{NQAF}P_{i,t}=\alpha_{1}+\theta_{1}\mathrm{DR}C_{i,t}+\beta_{1}X_{i,t}+\mu_{i}+\lambda_{t}+\varepsilon_{i,t}$$ (2)

$$\mathrm{GP}R_{i,t}=\alpha_{2}+\theta_{2}\mathrm{DR}C_{i,t}+\text{γNQAF}P_{i,t}+\beta_{2}X_{i,t}+\mu_{i}+\lambda_{t}+\varepsilon_{i,t}$$ (3)

The variables and parameters in Equations 2, 3 are defined as in Equation 1. $\text{NQAF}P_{i,t}$ denotes the mechanism variable, namely new quality agricultural productive forces.

Furthermore, based on the theoretical discussion above, this study uses fiscal support for agriculture (FSA) as the threshold variable and constructs a panel threshold model to examine whether the effect of digital rural construction on grain production resilience is nonlinear:

$$\begin{array}{l} \mathrm{GP}R_{i,t}=\theta_{0}+\theta_{1}\mathrm{DR}C_{i,t}I(\mathrm{FS}A_{i,t}\leq f_{1})\\ +\theta_{2}\mathrm{DR}C_{i,t}I(f_{1}<\mathrm{FS}A_{i,t}\leq f_{2})+\theta_{3}\mathrm{DR}C_{i,t}I(\mathrm{FS}A_{i,t}>f_{2})\\ +\theta_{4}X_{i,t}+\mu_{i}+\lambda_{t}+\varepsilon_{i,t} \end{array}$$ (4)

In Equation 4, $\theta_{1},\theta_{2}\;\text{and}\;\theta_{3}$ represent the effects of digital rural construction on grain production resilience under different ranges of fiscal support for agriculture. $\theta_{4}$ is the coefficient vector of the control variables, $\theta_{0}$ is the constant term, $f_{1}$ and $f_{2}$ are the estimated threshold values, and I(·) is an indicator function equal to 1 when the condition in parentheses is satisfied and 0 otherwise.

## 4 Results and analysis

### 4.1 Baseline regression results

The Hausman test statistic is 80.85 with a *p*-value of 0.0000, rejecting the null hypothesis that the random effects model is appropriate. Therefore, a two-way fixed effects model is used as the baseline specification to control for time-invariant provincial characteristics and common year shocks. As shown in column (1) of Table 5, digital rural construction is positively and significantly associated with grain production resilience (*α* = 0.578, *p* < 0.01). This result suggests that provinces with higher levels of digital rural construction tend to exhibit stronger grain production resilience. After control variables are progressively included in columns (2)–(6), the coefficient of digital rural construction remains positive and statistically significant, ranging from 0.807 to 0.886. In the fully specified model, the coefficient is 0.834, indicating that a one-standard-deviation increase in digital rural construction is associated with an increase of approximately 0.107 in grain production resilience. Given that the mean value of grain production resilience is 0.142, this association is economically meaningful. These findings support Hypothesis 1 and suggest that digital rural construction contributes to grain production resilience by improving information access, technology application, factor allocation, and risk-response capacity.

**Table 5:** Baseline regression results.

Variable	(1)	(2)	(3)	(4)	(5)	(6)
	GPR	GPR	GPR	GPR	GPR	GPR
Digital rural construction	0.578*** (0.204)	0.886*** (0.170)	0.882*** (0.171)	0.861*** (0.178)	0.807*** (0.170)	0.834*** (0.173)
Urbanization level		−0.865* (0.443)	−0.869* (0.438)	−0.865* (0.441)	−0.892** (0.425)	−0.891** (0.413)

Human capital level			0.007 (0.019)	−0.001 (0.019)	−0.014 (0.020)	−0.017 (0.020)
	
Traditional infrastructure construction				0.028 (0.043)	0.026 (0.041)	0.019 (0.042)
		
Economic development level					0.573* (0.335)	0.521 (0.330)
			
Agricultural mechanization intensity						0.073 (0.062)
				
Constant	0.556 (0.414)	0.602* (0.350)	0.583* (0.330)	0.549* (0.322)	0.978** (0.379)	0.986** (0.387)
Observations	360	360	360	360	360	360
*R*-squared	0.499	0.601	0.601	0.602	0.619	0.623
Year fixed effects	Yes	Yes	Yes	Yes	Yes	Yes
Province fixed effects	Yes	Yes	Yes	Yes	Yes	Yes

Robust standard errors are reported in parentheses. ***, **, and * indicate significance at the 1, 5, and 10% levels, respectively. The same applies to the following tables.

With respect to the control variables, the coefficient of urbanization is significantly negative in all specifications, suggesting a negative association between urbanization and grain production resilience in the sample. This result may be related to the conversion of agricultural land, the reallocation of rural labor, and changes in regional production structure during the urbanization process. However, it should also be interpreted with caution, as it may partly reflect regional heterogeneity or other unobserved factors. The coefficient of human capital is close to zero and statistically insignificant, indicating that no clear direct association with grain production resilience is observed in the current model. The coefficient of traditional infrastructure construction is also small and statistically insignificant, suggesting that its independent effect is not evident after controlling for other factors. Economic development is positive and significant at the 10% level in column (5), but becomes insignificant after all controls are included, implying that its relationship with grain production resilience may be mediated or absorbed by other regional characteristics. The coefficient of agricultural mechanization intensity is positive but statistically insignificant, indicating that the model does not provide sufficient evidence for an independent effect of mechanization on grain production resilience.

### 4.2 Robustness checks

To further assess the reliability and stability of the empirical results, this study conducts four robustness checks by re-estimating the baseline model.

First, municipalities directly under the central government are excluded. Beijing, Shanghai, Tianjin, and Chongqing differ substantially from other provinces in economic conditions and resource allocation, which may exert special influence on the results. To test the generalizability of the findings, these four municipalities are removed from the 30-province sample and the model is re-estimated. The results in column (1) of Table 6 show that the positive effect of digital rural construction on grain production resilience remains statistically significant, indicating that the baseline results are not driven by these special observations.

**Table 6:** Robustness checks.

Variable	(1)	(2)	(3)	(4)
	Excluding municipalities	Shortened time window	Replacing explanatory variable	Digital economy development level
Digital rural construction	0.749*** (0.120)	0.677*** (0.156)	0.605*** (0.155)	4.387*** (1.586)
Urbanization level	−0.031 (0.194)	−1.019** (0.408)	−0.806** (0.370)	−0.512 (0.312)
Human capital level	0.001 (0.014)	−0.023 (0.026)	−0.009 (0.020)	−0.019 (0.025)
Traditional infrastructure construction	0.072** (0.026)	0.014 (0.046)	0.012 (0.043)	0.081* (0.047)
Economic development level	0.205 (0.171)	0.449 (0.314)	0.662** (0.315)	0.530 (0.314)
Agricultural mechanization intensity	0.027 (0.038)	0.072 (0.050)	0.123* (0.065)	0.021 (0.070)
Constant	0.987*** (0.332)	0.598 (0.382)	0.554 (0.344)	−0.569 (0.349)
Observations	312	270	360	360
*R*-squared	0.766	0.616	0.642	0.605
Year fixed effects	Yes	Yes	Yes	Yes
Province fixed effects	Yes	Yes	Yes	Yes

Second, the sample period is shortened. To avoid short-term shocks and external disturbances caused by public emergencies such as the COVID-19 pandemic, which may have affected grain production order and agricultural operations, this study excludes observations from 2020 to 2022 and re-estimates the model using the relatively stable periods of 2012–2019 and 2023. Column (2) of Table 6 shows that digital rural construction continues to promote grain production resilience significantly, again confirming the robustness of the baseline conclusion.

Third, the core explanatory variable is replaced. To reduce potential measurement error arising from a single index construction method, this study recalculates the level of digital rural construction using principal component analysis and replaces the original core explanatory variable. As shown in column (3) of Table 6, the coefficient of the recalculated index remains positive and significant at the 1% level, consistent with the baseline regression. This indicates that the main conclusion is not sensitive to the specific measurement method used for digital rural construction.

Fourth, an extended measure of the core explanatory variable is used. To further examine robustness, this study uses the level of digital economy development as a substitute for digital rural construction. Unlike the previous test based on principal component analysis, this test adopts a broader measure of digital development to examine its effect on grain production resilience. Column (4) of Table 6 shows that the coefficient of digital economy development is 4.387 and is significantly positive at the 1% level, indicating that digital development still significantly enhances grain production resilience after the measure of the core explanatory variable is broadened.

### 4.3 Endogeneity test

The baseline regression may be affected by endogeneity, which could weaken the credibility of the estimated results. To address this concern, this study employs an instrumental variable approach. Specifically, it uses the interaction between the number of post offices in each province in 1984 and the lagged national number of Internet users as an instrument for digital rural construction. On the one hand, digital rural construction depends on communication infrastructure and digital networks. The number of provincial post offices in 1984 reflects the historical accumulation of regional communication infrastructure and the initial conditions for later digital development, satisfying the relevance requirement of the instrument. On the other hand, the number of post offices in 1984 is a historically predetermined endowment that is not affected by current regional economic development, while the lagged national number of Internet users is a macro-level exogenous indicator. Their interaction cannot directly affect current grain production resilience and can operate only indirectly through digital rural construction, satisfying the exogeneity condition.

Table 7 reports the two-stage least squares (2SLS) results. The first-stage regression shows that the coefficient of the instrumental variable is 0.241 and is significantly positive at the 1% level, indicating a strong correlation between the instrument and digital rural construction. The Kleibergen-Paap rk LM statistic is 38.663 (*p* = 0.0000), rejecting the null hypothesis of under-identification. The Kleibergen-Paap rk Wald F statistic is 67.301, which exceeds the Stock-Yogo critical value of 16.380 at the 10% maximal relative bias level, suggesting that weak instruments are unlikely to be a serious concern. Because this study uses one instrumental variable for one endogenous explanatory variable, the model is exactly identified; therefore, an overidentification test cannot be performed. The second-stage results show that the estimated coefficient of digital rural construction is 1.249 and is significantly positive at the 5% level. This indicates that, after accounting for potential endogeneity, digital rural construction remains positively associated with grain production resilience, which is consistent with the baseline regression results.

**Table 7:** Endogeneity analysis.

Variable	(1)		(2)	(3)
	First stage		Second stage	Lagged explanatory variable
Infrastructure network	0.241*** (0.069)			
		
Digital rural construction			1.249** (0.588)	0.922*** (0.210)
	
Economic development level	0.886*** (0.088)		−0.694 (0.644)	0.557 (0.333)
Urbanization level	−0.199** (0.090)		0.642*** (0.240)	−0.819* (0.402)
Agricultural mechanization intensity	0.058 (0.049)		−0.154 (0.148)	−0.013 (0.017)
Human capital level	−0.275*** (0.028)		0.427** (0.177)	0.004 (0.048)
Traditional infrastructure construction	0.106*** (0.034)		−0.091 (0.086)	0.073 (0.067)
Constant	−3.086*** (0.898)		−0.000 (0.116)	1.228*** (0.378)
Observations	360		360	330
LM statistic		38.663***		
Wald F statistic		67.301 [16.380]		
Controls	YES		YES	YES
*R*-squared	0.7668		0.1701	0.617

In addition, as a supplementary approach to addressing endogeneity, this study includes the one-period lag of digital rural construction in the model to alleviate potential reverse-causality concerns. The rationale is that the previous year’s level of digital rural construction is less likely to be directly affected by current grain production resilience than the contemporaneous variable. As shown in column (3) of Table 7, the coefficient of lagged digital rural construction is 0.922 and is significant at the 1% level. This result indicates that the positive association between digital rural construction and grain production resilience remains evident after the core explanatory variable is lagged by one period. However, the lagged-variable approach mainly helps reduce the possibility of reverse causality and cannot fully eliminate endogeneity arising from omitted variables or dynamic path dependence. Therefore, this result should be interpreted as auxiliary evidence from the endogeneity analysis rather than as definitive causal identification. Overall, the lagged-variable result is consistent with the instrumental-variable estimation and further supports the conclusion that digital rural construction contributes to grain production resilience.

### 4.4 Heterogeneity analysis

To further examine whether the effect of digital rural construction on grain production resilience varies across regions, this study conducts heterogeneity tests from two perspectives: eastern, central, and western China; and grain functional zones. The results are reported in Table 8. Existing research indicates that the effects of digital rural construction on food system resilience, agricultural production efficiency, and high-quality agricultural development are not homogeneous, but are constrained by regional development stages, agricultural foundations, and grain functional positioning (19, 38, 41).

**Table 8:** Heterogeneity analysis.

Variable	(1)	(2)	(3)	(4)	(5)	(6)
	Eastern region	Central region	Western region	Major producing areas	Major consuming areas	Balanced areas
Digital rural construction	0.533 (0.368)	0.430 (0.273)	0.888** (0.319)	0.542*** (0.130)	0.521 (0.318)	0.729* (0.361)
Constant	1.391* (0.644)	1.144** (0.441)	1.569** (0.697)	1.282*** (0.331)	1.289 (0.838)	0.990 (0.927)
Observations	132	96	132	156	84	120
R-squared	0.684	0.829	0.657	0.843	0.700	0.534
Year fixed effects	Yes	Yes	Yes	Yes	Yes	Yes
Province fixed effects	Yes	Yes	Yes	Yes	Yes	Yes

#### 4.4.1 Regional heterogeneity across eastern, central, and western China

Across eastern, central, and western China, the effect of digital rural construction on grain production resilience displays clear regional differences. The coefficient for western China is 0.888 and is significant at the 5% level. The coefficients for eastern and central China are 0.533 and 0.430, respectively; both are positive but statistically insignificant. These results indicate that the resilience-enhancing effect of digital rural construction is more prominent in western China.

A possible explanation is that western China has relatively weak agricultural infrastructure, information service systems, and market connectivity. Digital rural construction can compensate for these traditional agricultural constraints by improving information access, reducing transaction costs, and strengthening the organization of agricultural production, thereby generating a stronger marginal improvement in grain production resilience. Qin et al. (38) find that digital rural policies improve agricultural production efficiency more clearly in regions with lower levels of economic development and more rugged terrain, which is consistent with the western-region results in this study. By contrast, eastern China has a higher level of digital economy development, but agriculture accounts for a smaller share of the regional economy. The dividends of digitalization are therefore more concentrated in the secondary and tertiary sectors, and the marginal effect on grain production resilience is insignificant. Central China has a strong grain production foundation, but the deep integration of digital technologies with the grain production system still needs to be further released, so the positive effect of digital rural construction is not yet statistically significant.

#### 4.4.2 Heterogeneity across grain functional zones

From the perspective of grain functional zones, the grouped regression results show that digital rural construction is positively associated with grain production resilience in all three types of regions, but the statistical significance varies across groups. The coefficient for major grain-producing areas is 0.542 and is significant at the 1% level, while the coefficient for production-consumption balanced areas is 0.729 and is significant at the 10% level. The coefficient for major grain-consuming areas is also positive, at 0.521, but it is not statistically significant. These results suggest that the positive association between digital rural construction and grain production resilience is more evident in major grain-producing areas and production-consumption balanced areas, where grain production responsibilities and agricultural foundations are stronger. However, because this study does not conduct a formal between-group coefficient difference test, the results should be interpreted as evidence from grouped regressions rather than as proof of statistically significant differences across functional zones.

Specifically, major grain-producing areas play an important role in ensuring national grain supply security. Because they have large production scales and relatively solid agricultural foundations, digital rural construction may be more readily transformed into grain production resilience through production informatization, scale operation, and socialized service provision. Hao and Tan (19) show that digital rural construction significantly improves the resilience of China’s food system and that its effect is closely related to rural industrial integration and circulation digitalization. Research on grain green total factor productivity also suggests that major grain-producing areas with stronger agricultural foundations are more likely to sustain the enabling effect of agricultural socialized services (42). Production-consumption balanced areas undertake certain grain production functions while also facing regional supply–demand adjustment needs; digital technologies can promote coordination among production, circulation, and consumption, thereby enhancing the adaptability of grain production systems. Xu et al. (41) find that the impact of digital technology on grain production resilience varies across grain production-consumption regions, supporting the rationale for functional-zone grouping. By comparison, major grain-consuming areas have relatively limited local grain production scales and rely more on interregional circulation systems for food security. In the grouped regression results, the coefficient of digital rural construction in major grain-consuming areas is positive but not statistically significant. Therefore, this finding suggests that the positive association between digital rural construction and local grain production resilience is not clearly observed in this group, rather than proving that its effect is statistically weaker than that in other functional zones.

### 4.5 Mediation analysis

To test the transmission mechanism through which new quality agricultural productive forces link digital rural construction to grain production resilience, this study adopts the two-step mediation approach proposed by Jiang (40). The estimation results in Table 9 show that digital rural construction has a significant positive effect on new quality agricultural productive forces, with a coefficient of 0.070 at the 1% significance level. This suggests that digital rural construction promotes the formation of new quality agricultural productive forces through the diffusion of digital technologies, the embedding of data elements, and platform-based organizational connections, thereby upgrading agricultural laborers, means of production, and objects of labor. On this basis, this study further uses a bootstrap procedure with 500 replications to test the significance of the mediation effect. The results show that the indirect effect of digital rural construction on grain production resilience through new quality agricultural productive forces is 0.1918, with a 95% confidence interval of [0.0825, 0.3011]. Since the confidence interval does not include zero, the mediating effect is statistically significant at the 1% level. This indicates that new quality agricultural productive forces constitute an important transmission channel through which digital rural construction enhances grain production resilience. The core of new quality agricultural productive forces lies in transforming agricultural production through scientific and technological innovation and innovative factor allocation. It is not simply general technological progress, but a leap in the form of agricultural productive forces supported by high-quality laborers, new production tools, data resources, and modern business organizations (43, 44).

**Table 9:** Mediation-effect regression results.

Variable	(1)	(2)	(3)
	GPR	NQAFP	GPR
Digital rural construction	0.834*** (0.173)	0.070*** (0.017)	0.642*** (0.166)
Economic development level	0.521 (0.330)	0.006 (0.019)	0.504 (0.297)
Urbanization level	−0.891** (0.413)	−0.011 (0.014)	−0.860** (0.390)
Agricultural mechanization intensity	0.073 (0.062)	−0.001 (0.007)	0.075 (0.059)
Human capital level	−0.017 (0.020)	−0.002 (0.002)	−0.011 (0.017)
Traditional infrastructure construction	0.019 (0.042)	0.004 (0.002)	0.008 (0.040)
New quality agricultural productive forces			2.740*** (0.854)
Constant	0.986** (0.387)	0.240*** (0.027)	0.329 (0.363)
Observations	360	360	360
*R*-squared	0.604	0.709	0.630
Year fixed effects	Yes	Yes	Yes
Province fixed effects	Yes	Yes	Yes

Furthermore, after new quality agricultural productive forces are introduced into Equation 3, the direct effect of digital rural construction on grain production resilience remains significantly positive, with a coefficient of 0.642 at the 1% level. Meanwhile, the coefficient of new quality agricultural productive forces is 2.740 and is also significant at the 1% level. These results indicate that new quality agricultural productive forces play a significant transmission role in the relationship between digital rural construction and grain production resilience. The underlying logic is that digital rural construction not only improves information access and the technological application environment in agricultural production but also enhances grain production efficiency and risk response capacity by improving the digital literacy of agricultural laborers, promoting the use of intelligent agricultural machinery and agricultural data platforms, and optimizing the combination of land, capital, technology, and data. Once formed, new quality agricultural productive forces further strengthen the stable supply capacity, disaster response capacity, and continuous optimization capacity of the grain production system, thereby enhancing grain production resilience.

Compared with Equation 1, after new quality agricultural productive forces are included, the coefficient of digital rural construction decreases from 0.834 to 0.642 but remains significant. This suggests that new quality agricultural productive forces play a partial mediating role in the relationship between digital rural construction and grain production resilience. Thus, the impact of digital rural construction on grain production resilience is not limited to the direct empowerment of digital technologies; it also operates indirectly by transforming agricultural productive forces. In other words, digital rural construction promotes the development of new quality agricultural productive forces, optimizes the allocation of agricultural production factors, improves production efficiency, and strengthens agricultural risk response capacity, thereby indirectly improving grain production resilience. These results verify the mediating role of new quality agricultural productive forces and support Hypothesis 2.

### 4.6 Threshold effect test

Using fiscal support for agriculture as the threshold variable, this study applies the bootstrap method with 300 replications to test single, double, and triple-threshold effects. The threshold test results in Tables 10, 11 show that, when fiscal support for agriculture is used as the threshold variable, the effect of digital rural construction on grain production resilience is significantly nonlinear. Specifically, the F statistic for the single-threshold test is 100.21, with a *p*-value of 0.0000, and the F statistic is well above the 1% critical value of 83.3695. This indicates that the single-threshold effect passes the test at the 1% significance level, suggesting that fiscal support for agriculture plays a significant threshold role in the relationship between digital rural construction and grain production resilience.

**Table 10:** Threshold-effect test results.

Threshold variable	Number of thresholds	*F*-statistic	*p*-value	10% critical value	5% critical value	1% critical value
Fiscal support for agriculture	Single threshold	100.21	0.0000	41.8673	52.6355	83.3695
	Double threshold	47.04	0.0033	29.7350	34.0005	43.2368
	Triple threshold	8.62	0.8633	34.1200	38.8075	64.0334

**Table 11:** Threshold model estimation results.

Variable	Grain production resilience
DRC (FSA ≤ 0.0699)	0.548*** (0.070)
DRC (0.0699<FSA ≤ 0.1236)	0.176*** (0.057)
DRC (FSA > 0.1236)	−0.066 (0.067)
Control variables	Controlled
Constant	−0.072*** (0.015)
Observations	360
*R*-squared	0.674

The double-threshold test further shows an F statistic of 47.04 and a *p*-value of 0.0033. The *F* statistic is 47.04, which exceeds the 1% critical value of 43.2368, and the corresponding *p*-value is 0.0033. This indicates that the double-threshold effect is statistically significant at the 1% level. Fiscal support for agriculture therefore not only has a single-threshold effect but also displays a clear double-threshold pattern. The effect of digital rural construction on grain production resilience varies by stage as the level of fiscal support for agriculture changes.

The triple-threshold test is then conducted. The results show an F statistic of 8.62 and a *p*-value of 0.8633. Because the *p*-value exceeds 0.1 and the *F* statistic is below the 10% critical value of 34.1200, the triple-threshold effect is not statistically significant. Accordingly, this study selects the double-threshold model for subsequent estimation.

The threshold regression results show that fiscal support for agriculture has a significant nonlinear effect in the relationship between digital rural construction and grain production resilience, with two threshold values of 0.0699 and 0.1236. Specifically, when fiscal support is relatively low (FSA ≤ 0.0699), the coefficient of digital rural construction is 0.548 and is significant at the 1% level, indicating that the resilience-enhancing effect of digital rural construction is strongest when the fiscal support base is relatively weak. This may be because low-fiscal-support regions often face insufficient agricultural infrastructure, weak public service capacity, incomplete extension systems, and underdeveloped risk-prevention mechanisms. Digital rural construction can substantially compensate for shortcomings in information access, resource dispatch, technical services, and risk warning, leaving considerable room for marginal improvement. Previous studies show that fiscal expenditure affects agricultural growth through multiple pathways, including total factor productivity, improvements in machinery quality, and optimization of agricultural input structure; fiscal subsidies for agriculture can also affect grain total factor productivity through structural and technological effects (45, 46).

When fiscal support rises to a medium level (0.0699 < FSA ≤  0.1236), the coefficient of digital rural construction is 0.176 and remains significant at the 1% level. Compared with the low-fiscal-support interval, the coefficient is substantially smaller, indicating that the positive effect of digital rural construction on grain production resilience remains but weakens at the margin. One possible reason is that, as fiscal support increases, agricultural infrastructure, public services, extension systems, and risk-protection mechanisms have already improved to some extent. The scope for digital rural construction to continue filling basic gaps therefore becomes smaller, and its incremental effect shifts from gap-filling improvement to efficiency-oriented improvement. At this stage, whether digital rural construction can continue to generate benefits depends more on the alignment between fiscal funds and digital infrastructure, smart agricultural equipment, agricultural socialized services, and agricultural technological innovation. Research on the county-level digital economy and agricultural total factor productivity shows that digital technology conditions, organizational conditions, and environmental conditions may be complementary or substitutive; regions therefore need to choose differentiated pathways according to agricultural resource endowments and the foundation of the digital economy (15). In the sample, provinces whose fiscal support intensity exceeds 12.36% mainly include Inner Mongolia, Heilongjiang, Xinjiang, and several others.

When fiscal support exceeds the second threshold (FSA > 0.1236), the coefficient of digital rural construction is −0.066 and statistically insignificant. This indicates that, under a relatively high level of fiscal support, the positive association between digital rural construction and grain production resilience is no longer evident. This result should not be interpreted as evidence that digital rural construction has a negative effect, nor does it imply that fiscal support itself is ineffective. Rather, it suggests that simply increasing the intensity of fiscal support may not continuously strengthen the role of digital rural construction. One possible explanation is that, after fiscal support reaches a relatively high level, the marginal effect of digital rural construction depends more on the allocation structure, use efficiency, and compatibility of fiscal funds with digital agricultural development. If fiscal expenditure is not effectively linked to digital infrastructure utilization, agricultural technological innovation, socialized services, and risk-prevention capacity, its supporting role may be weakened. This interpretation is consistent with studies showing that agricultural productivity improvement depends not only on factor input expansion but also on the efficiency of factor allocation (47), and that the effect of agricultural subsidies depends on whether they promote land transfer, machinery service adoption, and improved production organization (48).

## 5 Discussion

### 5.1 Principal findings and relevance to nutrition and food security

This study examined how digital rural construction affects grain production resilience using panel data from 30 Chinese provinces from 2012 to 2023. The results show that digital rural construction is positively associated with grain production resilience, that new quality agricultural productive forces partially mediate this relationship, and that fiscal support for agriculture produces a nonlinear threshold effect. These findings extend the discussion of digital agriculture from productivity and rural development to the broader field of food and nutrition security. Although this study does not directly measure individual dietary intake or nutritional status, grain production resilience constitutes an upstream structural condition for food availability, staple-food price stability, and the capacity of food systems to protect nutrition security under climatic, market, and public-health shocks.

The positive effect of digital rural construction on grain production resilience suggests that digitalization can strengthen the production-side foundation of food security. Digital infrastructure, smart agriculture, digital finance, rural e-commerce, and digital governance improve information access, technology diffusion, resource allocation, and risk response in grain production. From a nutrition perspective, these improvements matter because disruptions in staple-food production can be transmitted to households through reduced food availability, higher food prices, and weakened purchasing power. A more resilient grain production system can therefore help stabilize the supply of basic dietary energy and create conditions for more reliable access to diverse and nutritious foods. This interpretation is consistent with the food-system resilience literature, which emphasizes that resilient food systems should maintain food and nutrition functions under shocks rather than merely restore aggregate output.

The heterogeneity results further show that the effect of digital rural construction is stronger in western China, major grain-producing areas, and production-consumption balanced areas. This finding has important implications for nutrition-sensitive food security policy. Western regions and production-consumption balanced regions often face weaker infrastructure, higher vulnerability to climate shocks, and larger marginal returns to digital public services. Strengthening digital rural construction in these areas may therefore improve the stability of local grain supply and reduce regional disparities in food availability. In major grain-producing areas, digital technologies can support scale-based production, disaster monitoring, machinery dispatch, and input management, thereby strengthening the supply base that supports national food security. By contrast, the insignificant effect in major grain-consuming areas indicates that digital rural policies in these regions may need to focus more on circulation, reserves, emergency logistics, and cross-regional coordination than on production-oriented digitalization alone.

The mediation results indicate that new quality agricultural productive forces are an important pathway through which digital rural construction enhances grain production resilience. Digital technologies do not improve resilience only by adding equipment or platforms to existing production systems. More importantly, they upgrade agricultural laborers, production tools, and factor allocation. This transformation can improve production efficiency, reduce resource waste, strengthen disaster response, and support greener production methods. For nutrition and sustainable-diet research, this mechanism is meaningful because stable and efficient grain production is a prerequisite for balancing food security, environmental sustainability, and dietary needs. In this sense, digital rural construction can be understood as a production-side intervention that supports the resilience and sustainability dimensions emphasized in contemporary food and nutrition research.

The threshold effect of fiscal support for agriculture shows that public investment shapes the extent to which digital rural construction can be translated into resilience gains. When fiscal support is relatively low, digital rural construction has the strongest positive effect, suggesting that digital infrastructure and digital services can compensate for basic weaknesses in agricultural information, services, and risk prevention. When fiscal support reaches a medium level, the positive effect remains significant but weakens. When fiscal support exceeds the second threshold, the effect becomes statistically insignificant. This result does not imply that agricultural fiscal support is unnecessary. Rather, it suggests that the nutrition-security value of fiscal policy depends on whether public funds are allocated to productive, resilient, and nutrition-relevant functions, such as disaster early warning, agricultural extension, digital public services, storage and logistics coordination, and stable staple-food supply, rather than being absorbed by repetitive projects or low-efficiency subsidies.

These findings also provide conditional insights for other developing countries seeking to advance agricultural digital transformation and strengthen food and nutrition security. Many developing countries face simultaneous challenges of climate vulnerability, weak agricultural infrastructure, limited fiscal capacity, and unstable food access. In contexts where staple foods remain central to household diets, improving the resilience of grain production can be an important component of nutrition-sensitive food-system governance. However, the Chinese experience should not be generalized mechanically. The effectiveness of digital rural construction depends on land systems, agricultural organization, market institutions, fiscal capacity, digital infrastructure, and the structure of public services. Future comparative research could examine whether similar digital-agricultural pathways operate in countries with different food systems, dietary structures, and institutional arrangements.

### 5.2 Policy implications

First, the foundation of digital rural construction should be continuously consolidated to improve digital support throughout the grain production process and to strengthen the production-side basis of nutrition security. Around key links in grain production, China should accelerate the development of rural broadband networks, the agricultural Internet of Things, remote sensing, agricultural big data platforms, and digital extension service systems. Data resources related to meteorological warning, pest and disease monitoring, machinery dispatch, input supply, market information, and grain reserve coordination should be interconnected and shared. For western China and regions with relatively weak agricultural foundations, priority should be given to closing gaps in digital infrastructure and public information services so that digital technologies are effectively embedded in risk identification, production decision-making, and post-disaster recovery in grain production.

Second, differentiated digital empowerment strategies should be implemented to improve the regional adaptability of digital rural construction policies. In major grain-producing areas, priority should be given to smart agriculture, precision irrigation, intelligent agricultural machinery, digital plant protection, and agricultural socialized service platforms, thereby improving the stability and shock resistance of scale-based grain production. In production-consumption balanced areas, digital technologies should be strengthened in the coordination of production, circulation, reserves, and consumption to enhance regional grain supply–demand adjustment capacity and reduce the risk of localized food-price volatility. In major consuming areas, digital platforms should play a greater role in interregional grain circulation, emergency reserve dispatch, and supply-chain coordination, avoiding the simple application of production-oriented digital rural construction models.

Third, new quality agricultural productive forces should be cultivated more rapidly to smooth the mechanism through which digital rural construction is transformed into grain production resilience. Agricultural technological innovation and digital technology diffusion should be used as key levers to promote the application of new means of production, such as intelligent agricultural machinery, agricultural sensors, drones, BeiDou navigation, and smart irrigation, in grain production. At the same time, digital literacy training for new agricultural business entities should be strengthened, and online extension services, agricultural data services, and socialized service systems should be improved to promote the coordinated upgrading of agricultural laborers, means of production, and objects of labor. Such measures can improve the capacity of grain producers to maintain stable production under shocks and provide more reliable support for food availability and dietary security.

Fourth, the structure of fiscal support for agriculture should be optimized according to the threshold characteristics identified in this study. Fiscal policy should shift from a focus on expenditure expansion to a focus on allocation quality, project relevance, and fund-use efficiency. In regions with relatively low fiscal support intensity, public investment should be directed toward basic weaknesses in agricultural infrastructure, digital information services, disaster warning systems, and agricultural public services, so that digital rural construction can more effectively fill gaps in grain production resilience. In regions with medium fiscal support intensity, fiscal funds should be better coordinated with smart agricultural equipment, agricultural technological innovation, digital extension services, and socialized service platforms to sustain the enabling effect of digital rural construction. In regions with relatively high fiscal support intensity, policy emphasis should shift toward performance evaluation, project-structure optimization, and the avoidance of repetitive construction or dependence on general subsidies. In this way, fiscal support can improve its compatibility with digital rural construction and promote the efficient transformation of public funds into stable, adaptive, and resilient grain production capacity that supports long-term food and nutrition security.

### 5.3 Limitations and future research

Although this study provides a relatively systematic analysis of the impact of digital rural construction on grain production resilience and its mechanisms, several limitations remain, especially in relation to its nutrition implications.

First, due to data availability, this study uses provincial panel data. Such data can reveal the overall macro-level relationship between digital rural construction and grain production resilience, but they cannot fully capture micro-level differences across counties, villages, households, and vulnerable groups. Future research could combine county-level data, household survey data, or microdata on agricultural operators to examine how digital rural construction affects household food access, dietary diversity, food expenditure, and nutrition-related vulnerability across different production actors, operation scales, and resource endowments.

Second, the measurement of digital rural construction and grain production resilience in this study mainly relies on composite evaluation systems constructed from statistical yearbooks and public data. Although this approach can reflect the overall levels of the two variables relatively comprehensively, the results may still be affected by indicator selection, statistical definitions, and weighting methods. With the development of smart agriculture, agricultural big data platforms, remote sensing, and digital extension services, future research could incorporate platform data, remote-sensing data, food-price data, and real-time agricultural production monitoring data to improve the precision and dynamic nature of measurement.

Third, this study focuses on the mediating role of new quality agricultural productive forces and the threshold effect of fiscal support for agriculture. However, the mechanisms through which digital rural construction affects food and nutrition security may be more complex. Future research could introduce variables such as agricultural socialized services, digital inclusive finance, agricultural insurance, scale-based farmland operation, agricultural extension systems, food circulation efficiency, and household food consumption to identify multiple transmission pathways. It could also combine macro-level production data with micro-level dietary and nutrition data to examine whether improvements in grain production resilience are translated into better diet quality and nutrition outcomes.

Fourth, this study is based on Chinese provincial samples and has implications for other developing countries, but its cross-regional and cross-country applicability still requires further testing. Future research could conduct cross-country comparisons or representative country case studies to compare the effects of digital agricultural policies under different levels of digital infrastructure, agricultural organization, fiscal support capacity, and dietary structure, thereby providing more targeted empirical evidence for agricultural digital transformation, global food security governance, and nutrition-sensitive development policy.

## Data Availability

The raw data supporting the conclusions of this article will be made available by the authors, without undue reservation.
